# The Role of Onabotulinum Toxin Type A in the Management of Chronic Non-migraine Headaches

**DOI:** 10.3389/fneur.2019.01009

**Published:** 2019-09-19

**Authors:** Cassie Jia, Scott Lucchese, Fang Zhang, Raghav Govindarajan

**Affiliations:** Department of Neurology, University of Missouri School of Medicine, Columbia, MO, United States

**Keywords:** Onabotulinum toxin type A (BoNTA), non-migraine headache, post-traumatic headache, primary headache, secondary headache, opioid use

## Abstract

**Objectives:** FDA has approved Onabotulinum toxin type A (BoNTA) for prophylactic treatment of chronic migraines. Recent studies have explored its potential new indications, like treating post-traumatic headaches.

**Patients and Methods:** This is a retrospective chart review of 717 patients, who had failed at least two prophylactic treatments and received BoNTA injections at University of Missouri Hospital from July 2014 to June 2017. Patient demographics, headache type, associated symptoms, prophylaxes tried were reported. Patient's pain severity (numeric pain scale) and frequency (number of headache days/month) pretreatment, at 6 months, and at 12 months were collected.

**Results:** For a single headache type, post-traumatic headaches showed reduction in headache pain severity at 6 months (2.9 ± 0.7) compared to pre-treatment (7 ± 0.7). Headache frequency for post-traumatic headaches was also reduced at 6 months (10.6 ± 2.3) and 12 months (5.1 ± 1.2) compared to pre-treatment (25 ± 1.8). For pseudotumor cerebri headaches, pain severity at pretreatment was 6.4 ± 0.6 compared to 2 ± 0.8 at 6 months, and headache days reduced at 6 months (9.8 ± 2.5) and 12 months (6 ± 4) compared to pretreatment (26 ± 2.9). Opioid use reduced by 67 ± 55.4 at 6 months and 133.3 ± 106.6 at 12 months in morphine equivalent units.

**Conclusions:** Onabotulinum toxin type A is effective in treating multiple types of chronic non-migraine headaches.

## Introduction

Headache is a common neurological symptom that has been recognized as a significant health issue. A study in 2008 found that 47% of the adult population had an active headache disorder globally (1). Recent data from 2012 National Health Interview Survey reported that 14.2% of US adults 18 or older had migraine or severe headaches in the previous 3 months (2). From 2009 to 2010, headache or headache pain became the fourth leading cause of visits to the emergency department (ED), and opioids were administered at 35% of these ED visits (2). Clearly, headaches account for loss of quality of life and work time and pose a significant burden to the individuals and healthcare system.

The clinical efficacy and safety of Onabotulinum toxin type A (BoNTA) in treating chronic migraine were previously demonstrated in the PREEMPT (Phase III Research Evaluating Migraine Prophylaxis Therapy) randomized clinical trial (3–5). The injection protocol of PREEMPT trials involved intramuscular administration of 155 U of BoNTA in 31 fixed-site, fixed dose injections across seven head/neck muscles every 12 weeks. These muscles include the corrugator, procerus, frontalis, temporalis, occipitalis, cervical paraspinal, and trapezius muscles. With a follow-the-pain strategy, an additional 40 U or maximum dose of 195 U can be administered into occipitalis, temporalis, and/or trapezius in one or both sides depending on location of reported predominant pain (4, 5). BoNTA's exact mechanism of action in headache relief is unknown but likely relates to inhibition of peripheral and central sensitization by attenuating neuropeptide and neurotransmitter exocytosis from peripheral sensory neurons (6). Experimental animal studies suggest that BoNTA can block calcitonin gene-related peptide and substance P release in response to noxious stimuli (6, 7).

Indications of BoNTA injections for other headache types are being explored. For instance, a randomized placebo-controlled trial of 300 patients demonstrated that BoNTA did not work as a prophylaxis for chronic tension type headaches, which may be explained by differences in protocol from original PREEMPT trials (4, 5, 8). In this study, patients received 10 intramuscular injections across 5 muscles (frontalis, sternocleidomastoid, upper trapezius, splenius capitis, and anterior temporalis) with the total BoNTA administered ranging from 50 U to 150 U (8). However, a 2015 retrospective chart review study performed on veterans with post-traumatic chronic migraine headaches demonstrated significant improvement in severity and frequency of headaches with BoNTA treatment (9). Currently, data is lacking in assessing the role of BoNTA in treating other chronic headaches. The purpose of this study is to evaluate the role of Onabotulinum toxin type A in treating multiple, chronic non-migraine headaches.

## Patients and Methods

**Figure 1:**
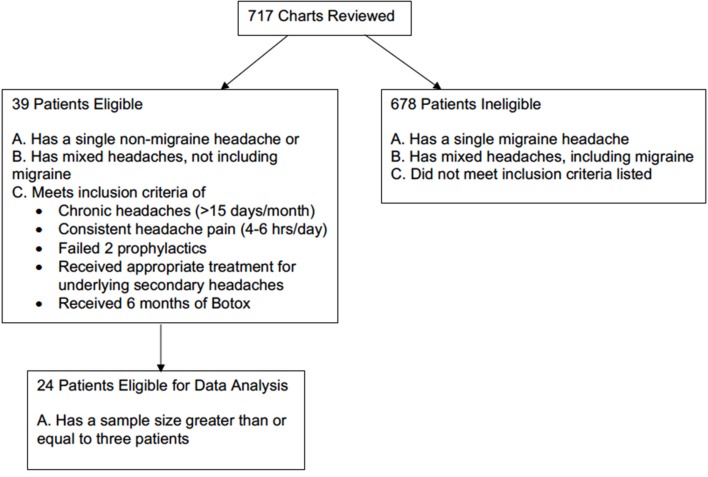
Subject enrollment selection criteria.

This is a retrospective chart review of 717 patients who had received BoNTA injections at University of Missouri Hospital from July 2014 to June 2017. To be eligible for inclusion, patients must have either a single non-migraines headache type or mixed headache types not including migraine that met the 3rd edition International Classification of Headache Disorders (ICHD-3) diagnostic criteria (10). Based on the headache type selection criteria, 59 patients qualified. They must also have chronic headaches, defined as having more than 15 headache days out of 30 days (4, 5). Additional qualifications included experiencing headaches 4–6 h per day and having received and failed the appropriate first-line treatments for the underlying causes of secondary headaches. For example, patients with obstructive sleep apnea headaches typically receive BoNTA treatments for persisting headaches and endorsing apnea-hypopnea index of <5 after having non-invasive CPAP ventilation for at least 2 months. Patients also needed to have failed at least two prophylactic treatments, meaning that they did not respond adequately to the prophylactic treatment either because targeted dose was not reached due to side effects or there was a lack of efficacy despite reaching the targeted dose. Given that patients took prophylaxis as needed and that this was a retrospective study, data on dosage changes of prophylactic treatment were not available. Patients were excluded from the study if they received <6 months of treatment or equivalent of two BoNTA injections. Previous studies suggested that 10% of a group of patients, who initially did not respond to first injection, did benefit from the second injections (11). A total of 39 patients received BoNTA injections as an off-label clinical treatment on these selection criteria. However, given the smaller sample size of several single and mixed non-migraine headaches, only 24 patients with four different non-migraine headache types were included in the final data analysis (Figure 1).

According to the PREEMPT injection paradigm of fixed-site and fix-dose injections, each patient received 155 U of BoNTA at 31 intramuscular injections across seven specific head/neck muscle areas every 3 months (4, 5). To avoid BoNTA spreading to undesired muscle groups, patients were instructed to not rub the injection sites.

Basic demographic data, including age, race, and sex, were collected from self-identified information available on electronic medical records (EMR). Previous clinical diagnoses of headache by physicians were categorized into primary or secondary headaches according to the ICHD-3 criteria (10). Reported associated symptoms were characterized as headache with migrainous features, including nausea or vomiting and photophobia and phonophobia (12). Number of prophylaxes taken before BoNTA treatment were recorded. Medications were considered prophylactic treatment if they were beta blockers, antidepressant (amitriptyline), anticonvulsant (divalproex and sodium valproate), topiramate, gabapentin, or lisinopril (13).

### Outcome Measures

Efficacy of BoNTA treatment was evaluated by multiple endpoints. Reduction in headache pain was assessed by self-reported pain on a numerical pain scale of 1–10 recorded on EMR pre-treatment and subsequent evaluations at 6 and 12 months (if applicable). Reduction in headache frequency was assessed by self-reported headache days in a month from patients' headache diaries and recorded on EMR pre-treatment and subsequent evaluations at 6 months and at 12 months (if applicable).

Descriptive statistics, including mean (SEM) and percentage, were used to describe the headache classification, associated clinical symptoms, and demographics reported in this study. Differences in outcomes were determined using one-way ANOVA multiple comparison analysis and Gabriel Students-Newman-Keuls post-test or paired *t*-tests and were considered significant when *p* < 0.05. All statistical analyses were performed using Sigma Plot (version 12) software (Systat Software).

## Results

### Patient Demographics

From July 2014 to June 2017, 717 patients received BoNTA injections after having failed at least two prophylactic treatments. Twenty-four patients with a single non-migraine headache type entered this study after having completed at least 6 months of BoNTA treatments and having a sample size large enough for statistical analysis. Baseline demographics were recorded (Table 1). Most patients were female (79%) and Caucasian (88%), and 96% of patients described their associated symptoms as headache with migrainous features: with characteristics of nausea or vomiting and photophobia and phonophobia (12). The mean patient age at baseline for female and male were 48 and 34 years old, respectively. Average number of prophylactic use prior to BoNTA injections was >4.

**Table 1:** Demographics and clinical characteristics.

**Characteristics**	**Patients (*n* = 24)**
**Age (years)**	
Female	47.58 ± 3.26
Male	34.40 ± 5.08
**Sex**, ***n*** **(%)**	
Female	19 (79%)
Male	5 (21%)
**Race**, ***n*** **(%)**	
White	21 (88%)
Black	2 (8%)
Hispanic	1 (4%)
**Social**, ***n*** **(%)**	
Smoking	4 (17%)
Alcohol	4 (17%)
Drugs	0 (0%)
**Migraine-like**	
Symptoms, *n* (%)	23 (96%)
Prophylactic use	4.17 ± 0.26

*Values are mean ± standard error of the mean*.

Out of the four non-migraine headache types studied, five patients used opioid to manage their chronic headache pain. Five data points were collected from these patients at 6 months, but only three patients had available data for 12 months. Opioid consumptions were measured in morphine equivalent units (MEU) at pretreatment, 6 months, and 12 months. Data on prophylactic treatments were not available for analysis.

### Headache Classification: Primary and Secondary

The causes of headaches are multi-factorial and can be classified according to their underlying etiologies. In this study, non-migraine headaches reported from 39 patients at baseline prior to BoNTA injections were characterized as primary or secondary headache(s) according to the ICHD-3 diagnostic criteria (10). Thirty-one patients reported having a single headache type, and eight patients had mixed headache types (Table 2). Only four non-migraine headache types had enough sample size (*N* > 3) to be included in the final data analysis; they include post-traumatic, obstructive sleep apnea, cervicogenic, and pseudotumor cerebri headaches.

**Table 2:** Headache Characterizations.

**Headache types**	**Patients (*n* = 39)**
Post-traumatic	11
Obstructive sleep apnea	4
Cervicogenic	4
Tension type	2
Pseudotumor cerebri	5
Chiari-malformation type 1	1
AV fistula	1
Orthostatic	2
Temporal arteritis	1
Post-traumatic & Obstructive sleep apnea	2
Post-traumatic & Tension type	1
Obstructive sleep apnea & Cervicogenic	2
Obstructive sleep apnea & Medication overuse	1
Obstructive sleep apnea & Chiari malformation type 1	1
Cervicogenic & Medication overuse	1

### Clinical Outcomes

**Figure 2:**
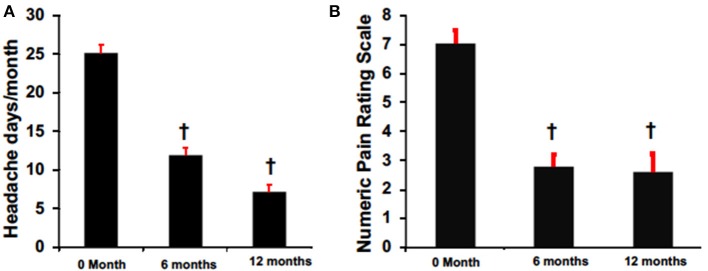
BoNTA treatment reduced headache days **(A)** and pain **(B)** in a time dependent manner. *n* = 16–24, ^*†*^*p* < 0.05 vs. 0 month.

Despite variable results observed among headache types, there was an overall mean reduction from baseline in headache frequency at 6 months (6 months: 11.9 ± 8.4 vs. pretreatment: 25.2 ± 1.3, *p* < 0.05) and 12 months (12 months 7.1 ± 1.8 vs. pretreatment: 25.2 ± 1.3, *p* < 0.05) in primary and secondary non-migraine headaches (Figure 2). Similarly, headache pain severity reduced from baseline compared to 6 months (6 months: 2.8 ± 0.4 vs. pretreatment: 7.0 ± 0.4, *p* < 0.05) and 12 months (12 months 2.6 ± 0.6 vs. pretreatment: 7.0 ± 0.4, *p* < 0.05) for all patients despite variable results observed among headache types (Figure 2B).

**Figure 3:**
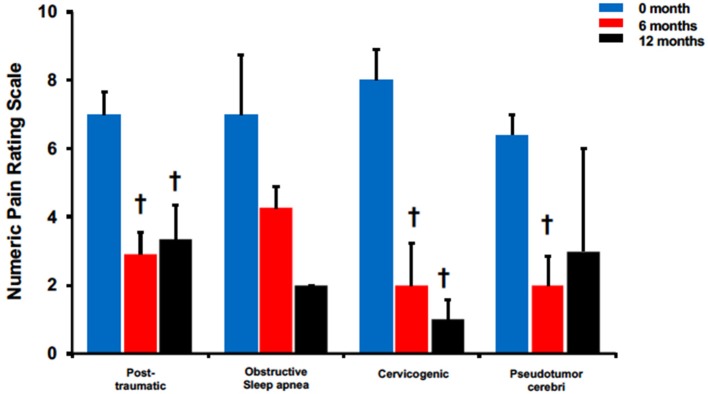
BoNTA treatment reduced pain severity for single non-migraine headache types in a time dependent manner. *n* = 2–11, ^*†*^*p* < 0.05 vs. 0 month.

The efficacy of BoNTA in reducing headache pain severity was evaluated in four non-migraine headache types (Figure 3). Post-traumatic headaches presented a significant reduction in headache pain severity at 6 months (6 months: 2.9 ± 0.7 vs. pre-treatment: 7 ± 0.7, *p* < 0.05) and 12 months (12 months: 3.3 ± 1 vs. pre-treatment: 7 ± 0.7, *p* < 0.05). Interestingly, pain reduction at 12 months was also less pronounced than at 6 months when compared to pretreatment. Similarly, compared to pretreatment, cervicogenic headaches had pain reduction at both 6 months (6 months: 2 ± 1.2 vs. pretreatment: 8 ± 0.9, *p* < 0.05) and 12 months (12 months: 1 ± 0.6 vs. pretreatment: 8 ± 0.9, *p* < 0.05). Pseudotumor cerebri also had a significant pain reduction at 6 months (6 months: 2 ± 0.8 vs. pretreatment: 6.4 ± 0.6, *p* < 0.05) but not at 12 months. Similarly, obstructive sleep apnea presented with a decreasing trend with non-significance at both 6 and 12 months.

**Figure 4:**
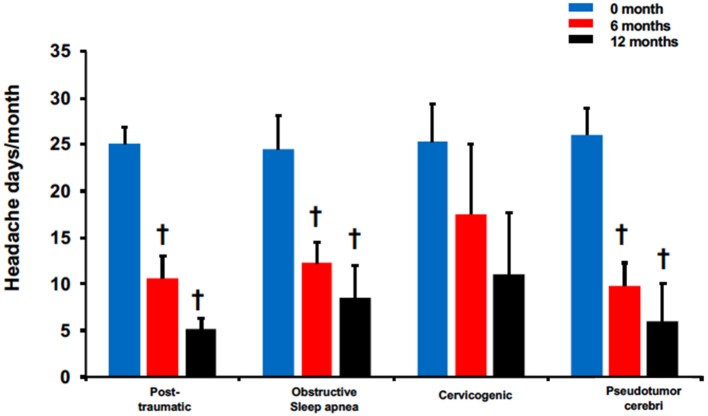
BoNTA treatment reduced headache days in single non-migraine headache types in a time dependent manner. *n* = 2–11, ^*†*^*p* < 0.05 vs. 0 month.

The effectiveness of BoNTA treatment was further assessed in these 4 headache types using headache frequency (Figure 4). All except for cervicogenic had significant reduction in headache days at 6 and 12 months. Patients with post-traumatic headaches reported 10.6 ± 2.3 at 6 months and 5.1 ± 1.2 at 12 months compared to 25 ± 1.8 pretreatment, *p* < 0.05. Similarly, patients with pseudotumor cerebri reported headache days at 26 ± 2.9 pretreatment compared to 9.8 ± 2.5 and 6 ± 4, *p* < 0.05 at 6 and 12 months, respectively. Interestingly, while patients with obstructive sleep apnea headaches did not experience significant pain reduction after BoNTA treatment (Figure 3), they did report a significant decrease in headache frequency at 6 months (6 months: 12.3 ± 2.3 vs. pretreatment: 24.5 ± 3.6, *p* < 0.05) and 12 months (12 months: 8.5 ± 3.5 vs. pretreatment: 24.5 ± 3.6, < 0.05). Only cervicogenic headaches had a decreasing trend with non-significance although they were associated with significant pain reduction at 6 and 12 months (Figure 3).

**Figure 5:**
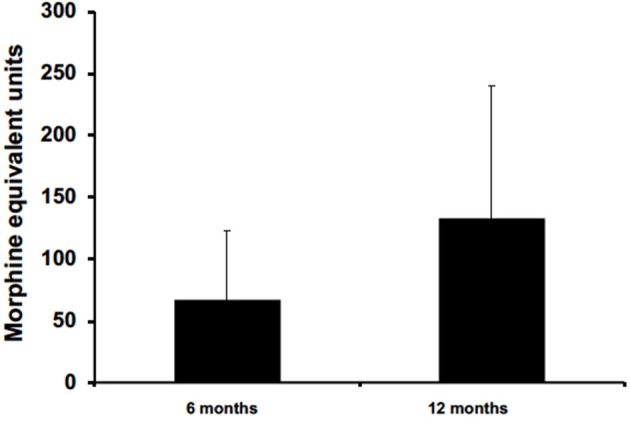
BoNTA treatment reduced opioid use in a time dependent manner (*n* = 3–5).

Five patients were prescribed opioids for chronic pain management. All had data available for 6 months, but only three patients had data available for 12 months. On average, data collected from these patients at 6 and 12 months reported opioid reduction by 67 ± 55.4 and 133.3 ± 106.6 in MEU, respectively (Figure 5). At 12 months for patients with post-traumatic headaches, one patient maintained opioid abstinence while the other continued to deescalate opioid intake and was able to stop. Another post-traumatic patient who had no reduction at 6 months was able to reduce intake by 5 MEU at 12 months. Two patients, with one having obstructive sleep apnea and the other with pseudotumor cerebri headaches, reported no reduction at 6 months and had no available data for 12 months.

## Discussion

We found that Onabotulinum toxin type A was effective in treating multiple types of chronic non-migraine headaches. Patients treated with BoNTA demonstrated a statistically significant decrease in both reported headache days and pain severity, suggesting a significant improvement in patient functionality and headache tolerability. Specifically, post-traumatic and cervicogenic headaches demonstrated significant reduction at 6 and 12 months compared to pre-treatment in terms of headache severity. In terms of headache frequency, patients with post-traumatic, obstructive sleep apnea, and pseudotumor cerebri reported significant reduction at 6 and 12 months compared to pre-treatment. Furthermore, there was a significant mean reduction comparing 6 and 12 months of treatment for cervicogenic headaches in terms of headache severity and for post-traumatic, obstructive sleep apnea, and pseudotumor cerebri headaches in terms of headache frequency. These findings suggest that the benefits of BoNTA were maintained and enhanced by additional treatments over time. It is not clear why post-traumatic headache pain reduction was less pronounced at 12 months than at 6 months when compared to pretreatment. It may be related to the small sampling size or time frame covered.

Our results are comparable to previous studies, where BoNTA have mixed results with different chronic non-migraine headaches. A randomize, double-blind, and placebo-controlled crossover study of 28 patients with chronic cervicogenic headache found no significant difference between BoNTA and placebo in terms of headache frequency and severity. These results may be partially explained by differences in injection protocol, where a total of 100 U of BoNTA were injected into occipital muscle, upper trapezius, splenius capitis, sternocleidomastoideus, and levator scapulae. (14). In comparison, a retrospective chart review of 64 patients with either primary or mixed chronic post-traumatic headaches reported benefits with BoNTA injections. In this study, 40 patients received the PREEMPT injection paradigm of 155 U BoNTA injections across 7 head/neck muscles, and additional injections for a total of 200 U were given for 11 patients with cervical dystonia and for 9 patients using the follow-the-pain technique. At the end, 41 patients reported feeling better (15). Additionally, a case study of a patient with chronic post-traumatic headache resistant to oral treatments also reported headache improvements 5 days after local injections of 22 U BoNTA into frontalis and corrugator supercilia muscles and remained symptoms free 10 days after treatment (16). Thus, the effectiveness of BoNTA in treating post-traumatic headaches suggested by our study is consistent with literature, and this has significant implication as headache is the most common symptom of both traumatic and mild concussion injury (17). Recent longitudinal studies reported the incidence of post-traumatic headaches to be 71% in moderate or severe traumatic brain injury and 91% in mild traumatic brain injury (18). Given that most research reporting benefits of BoNTA in post-traumatic headaches were prospective and retrospective studies (17), the effectiveness of BoNTA in treating post-traumatic headaches need to be further elucidated with randomized controlled studies.

Furthermore, the distribution of headache types demonstrated good correlation with patient demographics reported in this study. Migraine, for example, has a distinct female bias. Sacco et al. cited that the 1 year-migraine prevalence is 17% in women compared to 6% in men (19). Moreover, the lifetime incidence of migraine is reported to be 43% in women compared to 18% in men (19). Given that female patients made up 79% of this study population, the predominance of migraine headaches reported is consistent with female bias found in migraines. Interestingly, females were also found to be more likely to report any headache after 1 year of traumatic brain injury than males (74 vs. 63%) (20). In this study, 15 out of 27 patients reporting having at least one post-traumatic headaches were female. Besides gender correlation with certain headache types, current studies suggest a trend toward dose-dependent effect of tobacco smoking and other behavior stressors, like alcohol use, physical inactivity, obesity, and depression all working together to trigger headaches (21). In our cohort, 17% of patients reported smoking while 17% reported alcohol consumption.

Furthermore, the effectiveness of Onabotulinum toxin type A in treating multiple types of chronic, non-migraine headaches has several implications. First, because every patient must have failed at least two prophylactic treatments to qualify for BoNTA treatment, patients that were able to reduce their prophylaxis use during treatment suggest that BoNTA can reduce their associated side-effects. In this retrospective study, dosing changes of prophylactic medications were not available in the EMR, and future prospective study is necessary to quantify the dosage change of prophylactic treatments comparing BoNTA injections to pretreatment. Secondly, this study attempted to assess the role of BoNTA in impacting opioid dosing in chronic pain management. Our results from a sample size of nine patients suggest that BoNTA can reduce or stop opioid use or at least prevent dose escalation. For future study, it's possible to add the “follow-the-pain” protocol for additional BoNTA injections in patients whose chronic, non-migraine headaches did not respond initially to 12 months of BoNTA injection. In a prospective study of 155 patients receiving BoNTA injections for migraine and medication overuse headaches during a 2 years period, both 195 U and 155 U BoNTA injections were found to significantly reduce headache days, acute pain medication intake, and Headache Impact Test-6 scores compared to baseline. However, 195 U injection were superior in achieving all those measures compared to 155 U since the first injection and for all 2 years, suggesting a dose-dependent efficacy of BoNTA in treating migraine (22).

However, this study has several limitations. First, this is a retrospective chart review. Headache types were classified according to previous diagnosis made by different physicians. This raised the potential for lack of diagnosis standardization across physicians. For example, current literature suggests that post-traumatic headache, although primarily defined as a secondary headache by ICHD-3 diagnosis, may present with various primary headache phenotypes, with migraine being the most commonly reported followed by tension-type and cervicogenic headaches (23). Thus, it is possible that one physician characterized post-traumatic headaches with migraine phenotypes as a single secondary post-traumatic headache while another characterized the same headache as a secondary phenotype under the primary diagnosis of migraine and hence were excluded from this study.

The same limitation about characterizing post-traumatic headaches may have confounded the data from mixed headaches. Previously, a prospective study evaluating 212 patients within 1 week of mild traumatic brain injury found 91% cumulative incidence of post-traumatic headache at 1 year. Of those, up to 49% of headaches met criteria for migraine or probable migraine phenotype while up to 40% of headaches were characterized as tension-type (24). In this study, only patients with mixed headaches of migraine and post-traumatic etiologies demonstrated significant reduction in both pain severity and headache frequency at 6 and 12 months. Furthermore, post-traumatic headaches with obstructive sleep apnea and tension type phenotypes had significant pain reduction only at 6 months. These findings may be explained by these post-traumatic headaches having migraine phenotypes that were not reported. Thus, the effectiveness of BoNTA in treating mixed headaches with post-traumatic and migraine phenotypes may be confounded by the proven response of migraine headaches to BoNTA treatment alone. Similarly, another limitation to our study is that we had 51 out of 54 patients with “migraine-like” features as associated symptoms of their headaches. One can argue that patients with both non-migraine headaches and “migraine-like” features may have responded to BoNTA due to the action of BoNTA on the migraine components alone. However, isolating the effect of BoNTA to strictly headaches without migraine features is challenging in our small sample size of non-migraine headaches that are also predominantly associated with migraine-like features. This limitation can be modified with future prospective study, with exclusion criteria of patients with diagnosis of migraine in their mixed headaches and associated symptoms of migraine-like features.

## Conclusions

Onabotulinum toxin A can improve patients' headache tolerability for a number of chronic, non-migraine headaches. No unexpected treatment-related adverse events or reported side-effects were identified in any patient. Despite the limited sample size in this study, Onabotulinum toxin A may even play a role in reducing opioid use or at least prevent dose escalation in chronic headache pain management. Additional studies are needed to further elucidate the role of Onabotulinum toxin type A in chronic, non-migraine headache management.

## Data Availability Statement

The datasets generated for this study are available on request to the corresponding author.

## Ethics Statement

This studies involving human participants were reviewed and approved by MU Institutional Review Board. Written informed consent for participation was not required for this study in accordance with the national legislation and the institutional requirements.

## Author Contributions

CJ contributed to data collection and analysis and manuscript writing. SL contributed to provision of raw data and manuscript review. FZ contributed to provision of raw data. RG contributed to project design, data analysis, manuscript writing, and review.

### Conflict of Interest

The authors declare that the research was conducted in the absence of any commercial or financial relationships that could be construed as a potential conflict of interest.

## References

[1] Jensen R Stovner LJ. Epidemiology and comorbidity of headache. Lancet Neurol. (2008) 7:354–61. 10.1016/S1474-4422(08)70062-0 18339350

[2] Burch RC Loder S Loder E Smitherman TA. The prevalence and burden of migraine and severe headache in the United States: updated statistics from government health surveillance studies. Headache. (2015) 55:21–34. 10.1111/head.12482 25600719

[3] Lipton RB Rosen NL Ailani J DeGryse RE Gillard PJ Varon SF. OnabotulinumtoxinA improves quality of life and reduces impact of chronic migraine over one year of treatment: Pooled results from the PREEMPT randomized clinical trial program. Cephalalgia. (2016) 36:899–908. 10.1177/0333102416652092 27288354 PMC4959035

[4] Aurora SK Dodick DW Turkel CC DeGryse RE Silberstein SD Lipton RB. OnabotulinumtoxinA for treatment of chronic migraine: results from the double-blind, randomized, placebo-controlled phase of the PREEMPT 1 trial. Cephalalgia. (2010) 30:793–803. 10.1177/0333102410364676 20647170

[5] Diener HC Dodick DW Aurora SK Turkel CC DeGryse RE Lipton RB. OnabotulinumtoxinA for treatment of chronic migraine: results from the double-blind, randomized, placebo-controlled phase of the PREEMPT 2 trial. Cephalalgia. (2010) 30:804–14. 10.1177/0333102410364677 20647171

[6] Barbanti P Egeo G Fofi L Aurilia C Piroso S. Rationale for use of onabotulinum toxin A (BOTOX) in chronic migraine. Neurol Sci. (2015) 36(Suppl 1):29–32. 10.1007/s10072-015-2195-0 26017507

[7] Luvisetto S Gazerani P Cianchetti C Pavone F. Botulinum toxin type a as a therapeutic agent against headache and related disorders. Toxins. (2015) 7:3818–44. 10.3390/toxins7093818 26404377 PMC4591645

[8] Silberstein SD Göbel H Jensen R Elkind AH Degryse R Walcott JM. Botulinum toxin type A in the prophylactic treatment of chronic tension-type headache: a multicentre, double-blind, randomized, placebo-controlled, parallel-group study. Cephalalgia. (2006) 26:790–800. 10.1111/j.1468-2982.2006.01114.x 16776693

[9] Roka A Borgquist R Singh J. Coronary sinus lead positioning. Card Electrophysiol Clin. (2015) 7:635–47. 10.1016/j.ccep.2015.08.004 26596808

[10] Headache Classification Committee of the International Headache Society (IHS) The international classification of headache disorder 3rd edition. International Headache Society. Cephalalgia. (2013) 33:629–808. 10.1177/0333102413485658 23771276

[11] Dodick DW Turkel CC DeGryse RE Aurora SK Silberstein SD Lipton RB. OnabotulinumtoxinA for treatment of chronic migraine: pooled results from the double-blind, randomized, placebo-controlled phases of the PREEMPT clinical program. Headache. (2010) 50:921–36. 10.1111/j.1526-4610.2010.01678.x 20487038

[12] Peng KP Fuh JL Yuan HK Shia BC Wang SJ. New daily persistent headache: should migrainous features be incorporated? Cephalalgia. (2011) 31:1561–9. 10.1177/0333102411424620 21960650

[13] Modi S Lowder DM. Medications for migraine prophylaxis. Am Fam Physician. (2006) 73:72–8. 16417067

[14] Linde M Hagen K Salvesen Ø Gravdahl GB Helde G Stovner LJ. Onabotulinum toxin A treatment of cervicogenic headache: a randomised, double-blind, placebo-controlled crossover study. Cephalalgia. (2011) 31:797–807. 10.1177/0333102411398402 21300635

[15] Yerry JA Kuehn D Finkel AG. Onabotulinum toxin a for the treatment of headache in service members with a history of mild traumatic brain injury: a cohort study. Headache. (2015) 55:395–406. 10.1111/head.12495 25644249

[16] Lippert-Grüner M. Botulinum toxin in the treatment of post-traumatic headache - case study. Neurol Neurochir Pol. (2012) 46:591–4. 10.5114/ninp.2012.32109 23319227

[17] Conidi FX. Interventional treatment for post-traumatic headache. Curr Pain Headache Rep. (2016) 20:40. 10.1007/s11916-016-0570-z 27130542

[18] Lucas S. Posttraumatic headache: clinical characterization and management. Curr Pain Headache Rep. (2015) 19:48. 10.1007/s11916-015-0520-1 26280569

[19] Sacco S Ricci S Degan D Carolei A. Migraine in women: the role of hormones and their impact on vascular diseases. J Headache Pain. (2012) 13:177–89. 10.1007/s10194-012-0424-y 22367631 PMC3311830

[20] Hoffman JM Lucas S Dikmen S Braden CA Brown AW Brunner R. Natural history of headache after traumatic brain injury. J Neurotrauma. (2011) 28:1719–25. 10.1089/neu.2011.1914 21732765 PMC3172878

[21] Taylor FR. Tobacco, nicotine, and headache. Headache. (2015) 55:1028–44. 10.1111/head.12620 26140522

[22] Negro A Curto M Lionetto L Martelletti P. A two years open-label prospective study of OnabotulinumtoxinA 195 U in medication overuse headache: a real-world experience. J Headache Pain. (2015) 17:1. 10.1186/s10194-016-0591-3 26792662 PMC4720620

[23] Minen MT Boubour A Walia H Barr W. Post-concussive syndrome: a focus on post-traumatic headache and related cognitive, psychiatric, and sleep issues. Curr Neurol Neurosci Rep. (2016) 16:100. 10.1007/s11910-016-0697-7 27709555

[24] Lucas S Hoffman JM Bell KR Dikmen S. A prospective study of prevalence and characterization of headache following mild traumatic brain injury. Cephalalgia. (2014) 34:93–102. 10.1177/0333102413499645 23921798

